# The genetic landscape of crystallins in congenital cataract

**DOI:** 10.1186/s13023-020-01613-3

**Published:** 2020-11-26

**Authors:** Vanita Berry, Alex Ionides, Nikolas Pontikos, Michalis Georgiou, Jing Yu, Louise A. Ocaka, Anthony T. Moore, Roy A. Quinlan, Michel Michaelides

**Affiliations:** 1 Department of Genetics, UCL Institute of Ophthalmology, University College London, 11-43 Bath Street, London, EC1V 9EL UK; 2 Moorfields Eye Hospital NHS Foundation Trust, London, EC1V 2PD UK; 3 Ophthalmology Department, University of California School of Medicine, San Francisco, CA 94158 USA; 4 Nuffield Department of Clinical Neurosciences, University of Oxford, Oxford, OX3 9DU UK; 5 GOSgene, Genetics and Genomic Medicine, UCL Great Ormond Street Institute of Child Health, London, WC1N 1EH UK; 6 Department of Biosciences, University of Durham, Upper Mountjoy Science Site, Durham, DH1 3LE UK

**Keywords:** Autosomal dominant congenital cataract, Next generation sequencing, Crystallins

## Abstract

**Background:**

The crystalline lens is mainly composed of a large family of soluble proteins called the crystallins, which are responsible for its development, growth, transparency and refractive index. Disease-causing sequence variants in the crystallins are responsible for nearly 50% of all non-syndromic inherited congenital cataracts, as well as causing cataract associated with other diseases, including myopathies. To date, more than 300 crystallin sequence variants causing cataract have been identified.

**Methods:**

Here we aimed to identify the genetic basis of disease in five multi-generation British families and five sporadic cases with autosomal dominant congenital cataract using whole exome sequencing, with identified variants validated using Sanger sequencing. Following bioinformatics analysis, rare or novel variants with a moderate to damaging pathogenicity score, were filtered out and tested for segregation within the families.

**Results:**

We have identified 10 different heterozygous crystallin variants. Five recurrent variants were found: family-A, with a missense variant (c.145C>T; p.R49C) in *CRYAA* associated with nuclear cataract; family-B, with a deletion in *CRYBA1* (c.272delGAG; p.G91del) associated with nuclear cataract; and family-C, with a truncating variant in *CRYGD* (c.470G>A; W157*) causing a lamellar phenotype; individuals I and J had variants in *CRYGC* (c.13A>C; T5P) and in *CRYGD* (c.418C>T; R140*) causing unspecified congenital cataract and nuclear cataract, respectively. Five novel disease-causing variants were also identified: family D harboured a variant in *CRYGC* (c.179delG; R60Qfs*) responsible for a nuclear phenotype; family E, harboured a variant in *CRYBB1* (c.656G>A; W219*) associated with lamellar cataract; individual F had a variant in *CRYGD* (c.392G>A; W131*) associated with nuclear cataract; and individuals G and H had variants in *CRYAA* (c.454delGCC; A152del) and in *CRYBB1* (c.618C>A; Y206*) respectively, associated with unspecified congenital cataract. All novel variants were predicted to be pathogenic and to be moderately or highly damaging.

**Conclusions:**

We report five novel variants and five known variants. Some are rare variants that have been reported previously in small ethnic groups but here we extend this to the wider population and record a broader phenotypic spectrum for these variants.

## Background

Familial cataract is a clinically and genetically heterogeneous disease with an incidence of 1–6/10,000 live births in developed countries and 5–15/10,000 births in developing countries [1, 2]. Congenital cataract can occur in isolation or as part of other systemic disorders. Nearly half of inherited cataracts are autosomal dominantly inherited, followed by autosomal recessive and X-linked. Congenital cataracts are phenotypically heterogeneous due to various spatiotemporal insults experienced during lens development. The most common phenotype is nuclear cataract, followed by posterior polar, total, lamellar, blue-dot, coralliform, anterior polar, pulverulent, cortical, complete, and finally polymorphic [3, 4]. Approximately 50 disease-causing genes have been identified to date associated with isolated cataract. Pathogenic variants have been identified in genes encoding many different proteins including, water channel proteins (MIP/AQP0) which regulate water transport; membrane gap junction proteins (CX50, CX46); cytoskeletal proteins (BFSP1, BFSP2, VIM) which stabilise the plasma membrane and the fibre cells themselves; transcription factors including (PAX6, PITX3, FOXE3, and MAFA); genes with various functions (EPHA2, FYCO1, TDRD7), and the intracellular lens proteins, the crystallins (https://cat-map.wustl.edu/) [5], iSyTE version 2.0).

Crystallins—α, β and γ constitute approximately 90% of all lens proteins and the major soluble proteins in the newborn lens. They are also responsible for the refractive index (RI) gradient of the lens, but they can also be membrane-associated and this increases with age [6]. Alpha-crystallins are molecular chaperones and members of the small heat shock protein family, protecting lens proteins from aggregation and therefore preventing lens opacification [7]. The α-crystallin comprises two subunits (αA polypeptide and αB polypeptide) encoded by *CRYAA* and *CRYAB*, respectively [7–10]. So far, fifty-seven (18.5%) disease causing variants in *CRYAA* are responsible for both autosomal dominant (AD) and autosomal recessive (AR) cataract. *CRYAA* is mainly expressed in the lens, but is also present in the retina and cornea. *CRYAB* is expressed in the lens epithelial cells and also in many other tissues such as the retina, skeletal muscle, heart, kidney and brain [11–14]. Sequence variants in *CRYAB* cause not only cataract, but also cardiomyopathies. Specific enhancers regulate *CRYAB* expression in lens and heart tissues [15]. Berry and colleagues, found the first dominant heterozygous *CRYAB* variant in a British pedigree with posterior polar cataract [16]. To date, twenty-two sequence variants (7.1%) have been reported for *CRYAB,* linked to both AD and AR cataract. The βγ-crystallins are derived by gene duplication, comprising four homologous Greek key motifs arranged into two domains. The β-crystallin family comprises three acidic (A) and three basic (B) forms encoded by the genes, *CRYBA1*, *CRYBA2*, *CRYBA4* and *CRYBB1*, *CRYBB2*, *CRYBB3* respectively. To date, a large number of disease-causing variants have been found: in βA1 (thirty-seven), βB2 (forty-four), βB1 (twenty-five), βA4 (eight), βB3 (eight) and βA2 (three). The γ-crystallins are encoded by the γ-gene cluster encompassing genes γA (*CRYGA*) to γD (*CRYGD*). Fewer sequence variants have been identified in γA (two) and γB (three) as compared to γC (thirty-five) and γD (fifty-six). Interestingly, most of the variants in the *CRYGC* and *CRYGD* genes cause autosomal dominant nuclear and coralliform cataract phenotypes. There is a single γS-crystallin gene (*CRYGS*) and its variants (eight) are linked to AD cataract, but with a broad phenotypic spectrum.

**Fig. 1:**
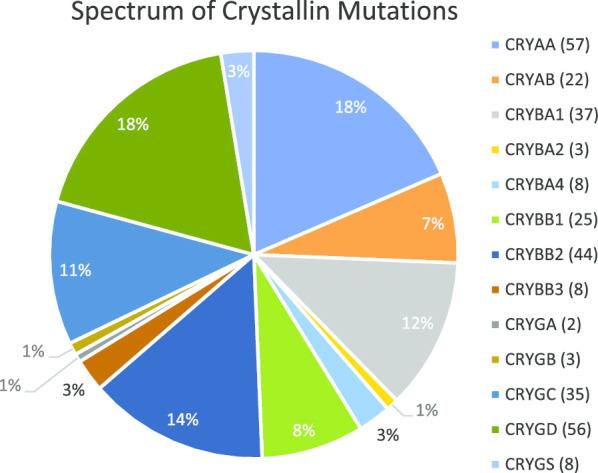
Frequency pie charts showing spectrum of cataract-causing crystallin variants. Total number of 308 disease-causing variants to date (novel and recurrent) are shown in 13 crystallins expressed in lens. (https://cat-map.wustl.edu/)

To date, 308 disease-causing variants have been found in total in the crystallins, accounting for nearly 23.0% of all inherited cataract variants (Fig. 1) [5, 17]. In this study, we have undertaken whole-exome sequencing (WES) in order to identify pathogenic variants underlying autosomal dominant congenital cataract (ADCC) in five large families of British origin and five sporadic cases from our ADCC panel.

## Methods

### Phenotyping

The patients studied were identified through the proband attending the Genetic Service at Moorfields Eye Hospital, London, UK. The study protocol adhered to the Tenets of the Declaration of Helsinki and was approved by UCL research ethics committee, (project ID -4817/001). All the family members participating in this study gave written informed consent and underwent full ophthalmic examination, including slit lamp examination. All affected individuals from five families and 5 isolated cases were diagnosed as having an isolated congenital cataract as described below.

#### Whole exome sequencing and bioinformatics analysis

Genomic DNA was extracted from EDTA sequestered blood samples using the Nucleon II DNA Extraction Kit (Scotlab Bioscience, Strathclyde, Scotland, UK). The DNA samples were sequenced at Macrogen Europe. Exon capture and target enrichment was performed using the SureSelectXT Human All Exon V6 post, (Agilent, Santa Rosa, CA, USA). Paired-end sequencing was performed on an Illumina Hiseq 2500 high-throughput sequencer, generating mean exome coverage of 50×. Raw data in fastq format was analysed using the Phenopolis bioinformatics platform [18]. The short-read sequence data were aligned to the GRCh37/hg19 human reference sequence using Burrows-Wheeler Aligner (BWA-MEM) and then marked duplicates with GATK*’s MarkDuplicates. Variants and indels were called according to GATK (version 3.5.0) best practices (joint variant calling followed by variant quality score recalibration). The moderately or highly damaging variants were then annotated using the Variant Effect Predictor (VEP) [19]. Variants with a sequencing depth of less than 20 × were filtered out. Variants were then filtered to only contain novel variants which were absent in public control databases Kaviar (https://db.systemsbiology.net/kaviar/) [20] and Genome Aggregation Database (gnmAD, https://gnomad.broadinstitute.org/) or rare variants (GnomAD allele frequency < 0.0001). Recurrent mutations were identified from 356 known cataract genes (https://cat-map.wustl.edu/) and predicted to be moderately or highly damaging (CADD>15). The filtered variants were then ordered on CADD score with the highest at the top. Further bioinformatic validations were done on the varsome platform (varsome.com).

**Fig. 2:**
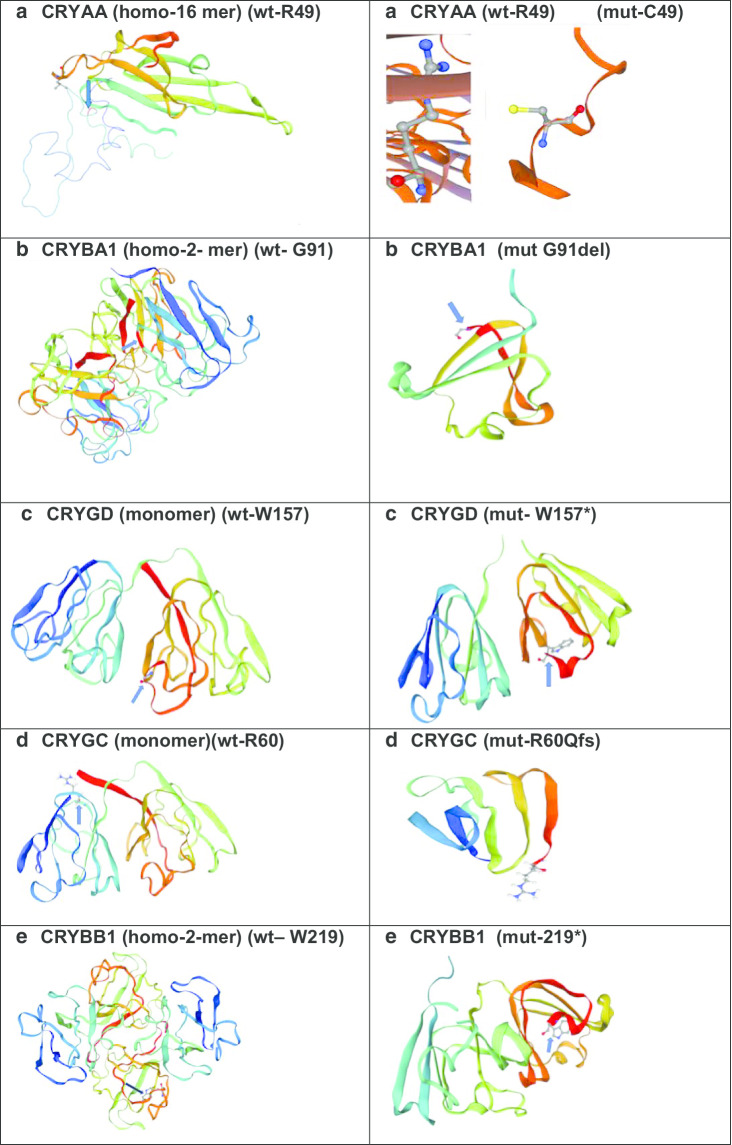

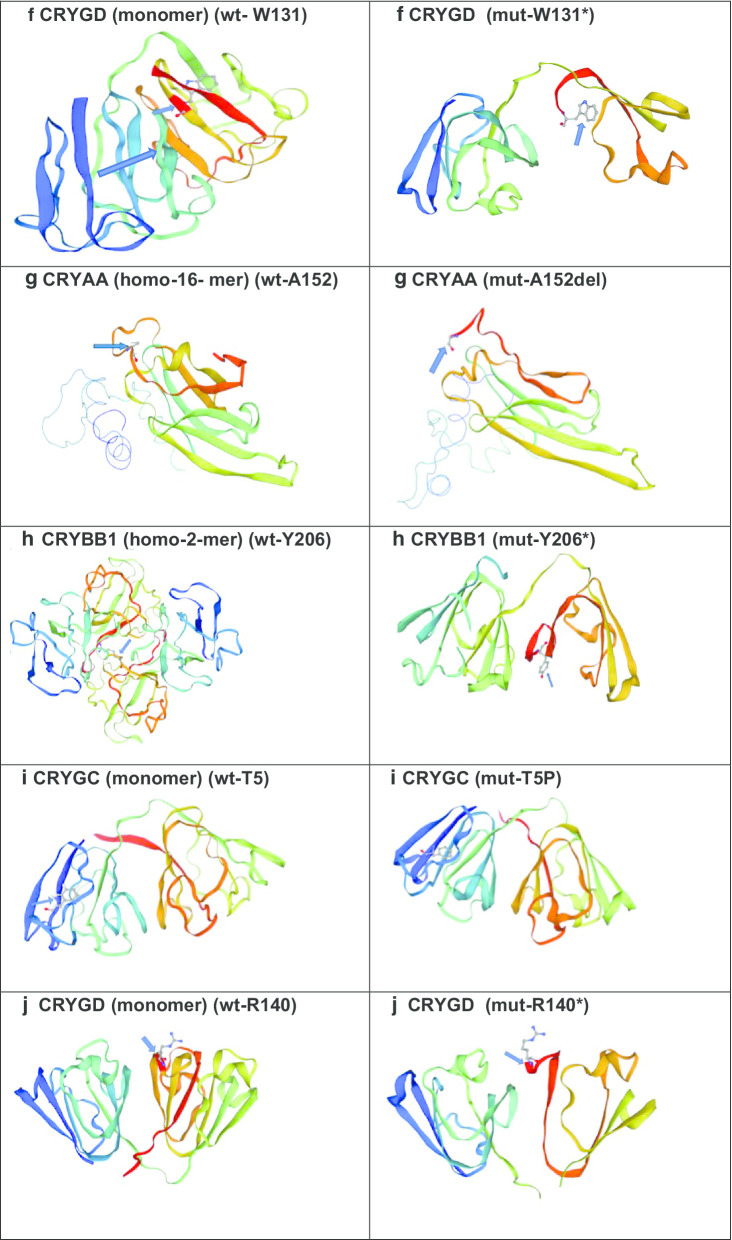
Structural view of Crystallins: (https://swissmodel.expasy.org/repository/uniprot/) **a** *CRYAA—*wild-type and missense mutant amino acid at position 49 (Arginine); **b** *CRYBA1*—wild-type and indel mutant amino acid at position 91(Glycine); **cCRYGD** wild-type and mutant stop codon at amino acid position 157 (Tryptophan); **d** *CRYGC*—wild-type and mutant frame-shift variant at amino acid position 60 (Arginine); **e** *CRYBB1*—wild-type and mutant stop codon amino acid at position 219 (Tryptophan); **f** *CRYGD*–wild-type and mutant stop codon amino acid at 131 (Tryptophan); **g** CRYAA—wild-type and mutant indel variant at amino acid position 152 (Alanine); **h** CRYBB1—wild-type and mutant stop codon amino acid at 206 (Tyrosine); **i** *CRYGC*—wild-type and missense mutant amino acid at position 5 (Threonine) and **j** CRYGD—wild-type and mutant stop codon amino acid at 140 (Arginine)

The protein structure of crystallins was analysed using SWISSMODEL in CRYAA, CRYGD,

CRYGC, CRYBA1 and CRYBB1 (Fig. 2):

CRYAA-Wt/homo-16-mer (https://swissmodel.expasy.org/interactive/5andJH/templates/);

CRYAA-Mut/(R49C) (https://swissmodel.expasy.org/interactive/Mw23CN/templates/);

CRYAA-Mut/(A152 del) (https://swissmodel.expasy.org/interactive/UQKAJV/templates/);

CRYGD-Wt-monomer (https://swissmodel.expasy.org/interactive/Hfak2y/templates/);

CRYGD-Mut/R140 (https://swissmodel.expasy.org/interactive/K8jQJF/templates/);

CRYGD-Mut/W131* (https://swissmodel.expasy.org/interactive/Vg8tcE/models/);

CRYGD Mut/W157* (https://swissmodel.expasy.org/interactive/H7Kg9S/models/);

CRYGC-Wt (https://swissmodel.expasy.org/repository/uniprot/A0A0X8GLL6);

CRYGC-Mut/T5P (https://swissmodel.expasy.org/interactive/JRTDhS/models/);

CRYGC-R60Qfs (https://swissmodel.expasy.org/interactive/JsUkC2/models/);

CRYBA1/Wt (https://swissmodel.expasy.org/repository/uniprot/P05813);

CRYBA1-Mut/G91del (https://swissmodel.expasy.org/interactive/NCCBgG/);

CRYBB1-Wt (https://swissmodel.expasy.org/repository/uniprot/P53674);

CRYBB1-Mut/Y206*(https://swissmodel.expasy.org/interactive/HXyC6C/models/);

CRYBB1-Mut/W219* (https://swissmodel.expasy.org/interactive/3cEHL5/).

### Sanger sequencing

Direct Sanger sequencing was performed to validate the variant identified by whole exome sequencing. Genomic DNA was amplified by PCR using GoTaq 2X master mix (AB gene; Thermo Scientific, Epsom, UK) and *CRYAA, CRYBA1, CRYBB1, CRYGA, CRYGC* and *CRYGD* -specific primers designed with https://bioinfo.ut.ee/primer3-0.4.0/

PCR conditions were as follows: 94 °C for 5 min of initial denaturation followed by 30 cycles of amplification of 30 s at 94 °C denaturing, 30 s at 60 °C annealing, and 45 s at 72 °C for extending. After cleaning, the PCR products were reacted with BigDye Terminator v3.1, they were run on ABI 3730 Genetic Analyzer (both from Applied Biosystems, Foster City, CA, USA) and analysed using SeqMan Pro (version 8.0.2 from DNASTAR) sequence analysis. After validating the variant, segregation was performed in all the available family members.

## Results

In this study we have investigated five European families with autosomal dominant congenital cataract, family A–E, and five isolated individuals F–J (Table 1).

**Table 1:** Crystallin disease-causing variants implicated in ADCC families/isolated cases in present study

Family	Variant	Gene	HGVSc	HGVSp	Phenotype	CADD	GERP	Mutation taster/verdict
A	*Chr21-44589354*	*CRYAA*	c.145C>T	R49C	Nuclear/lamellar	32.00	4.88	Disease causing-0.81/likely pathogenic/recurrent
B	*Chr17-27579135*	*CRYBA1*	c.272delGAG	G91del	Nuclear	19.47	5.88	Disease causing-0.81/pathogenic/recurrent
C	*Chr2-208986452*	*CRYGD*	*c.470G*>*A*	W157*	Pulverulent	39.00	4.25	Disease causing-0.81/pathogenic/recurrent
D	*Chr2-208994238*	*CRYGC*	c.179delG	R60Qfs*43	Nuclear	32.00	4.98	Disease causing-0.81/pathogenic/novel
E	*Chr22-26995557*	*CRYBB1*	c.656G>A	W219*	Lamellar	43.00	4.21	Disease causing-0.81/pathogenic/novel
F	*Chr2-208986530*	*CRYGD*	c.392G>A	W131*	Nuclear	40.00	4.25	Disease causing-0.81/pathogenic/novel
G	*Chr21-44592322*	*CRYAA*	c.454delGCC	A152del	Congenital cataract	15.18	3.78	Disease causing-0.81/likely pathogenic/novel
H	*Chr22-26995595*	*CRYBB1*	c.618C>A	Y206*	Congenital cataract	38.00	4.21	Disease causing-0.81/pathogenic/novel
I	*Chr2-208994404*	*CRYGC*	c.13A>C	T5P	Congenital cataract	24.80	4.96	Disease causing-0.81/likely pathogenic/recurrent
J	*Chr2-208986504*	*CRYGD*	c.418C>T	R140*	Nuclear cataract	36.00	4.25	Disease causing-0.81/pathogenic/recurrent

*CADD* combined annotation dependent depletion, *GERP* genomic evolutionary rate profiling

### Families

**Fig. 3:**
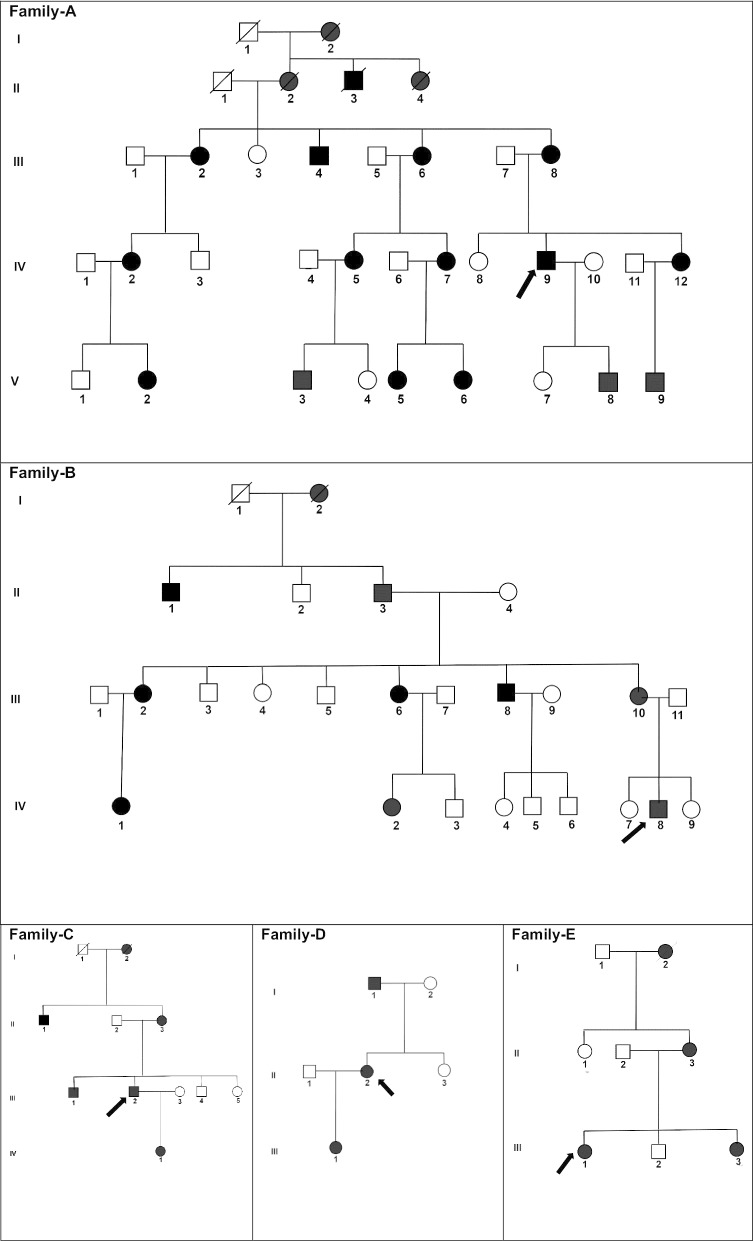
**a** Family A: Abridged pedigree with nuclear cataract; **b** Family B: Abridged pedigree with nuclear cataract; **c** Family C: Abridged pedigree with pulverulent cataract; **d** Family D: Abridged pedigree with nuclear cataract; **e** Family E: Abridged pedigree with lamellar cataract. The diagonal line indicates a deceased family member. Squares and circles symbolize males and females, respectively. Open and filled symbols indicate unaffected and affected individuals, respectively. The arrow indicates the family members who participated in the WES analysis. All the members available in the family were sequenced to show the segregation

**Fig. 4:**
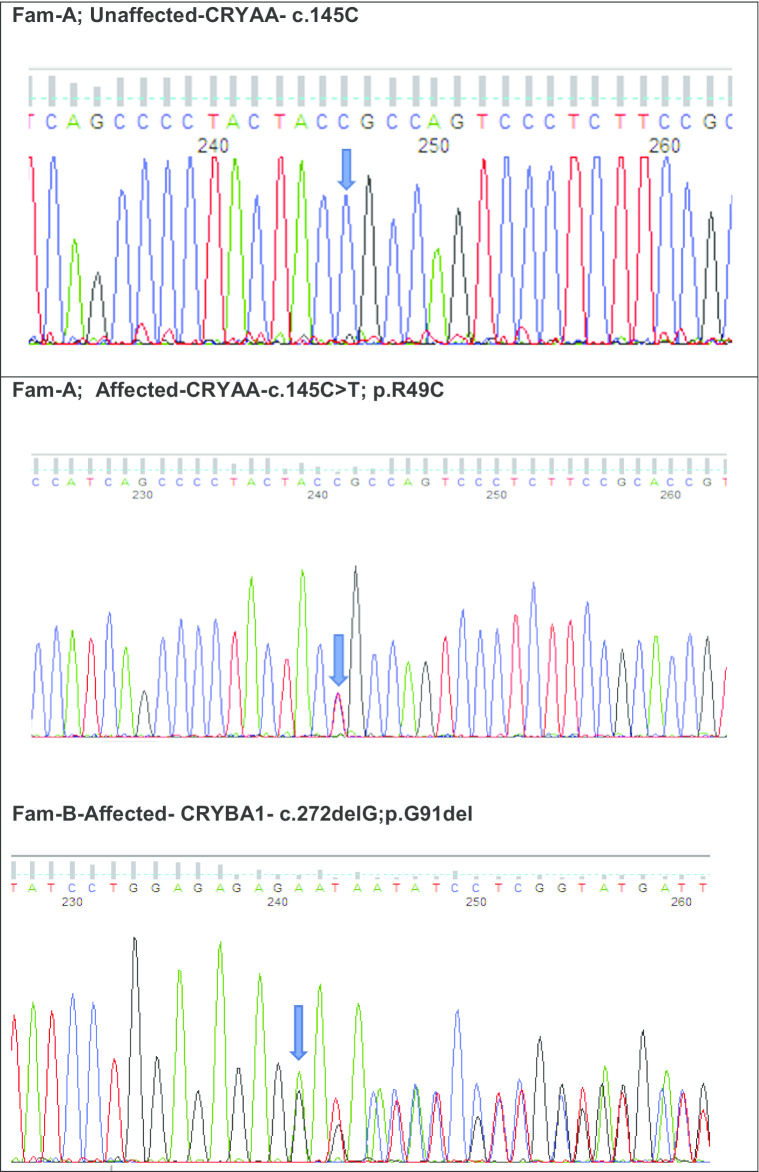

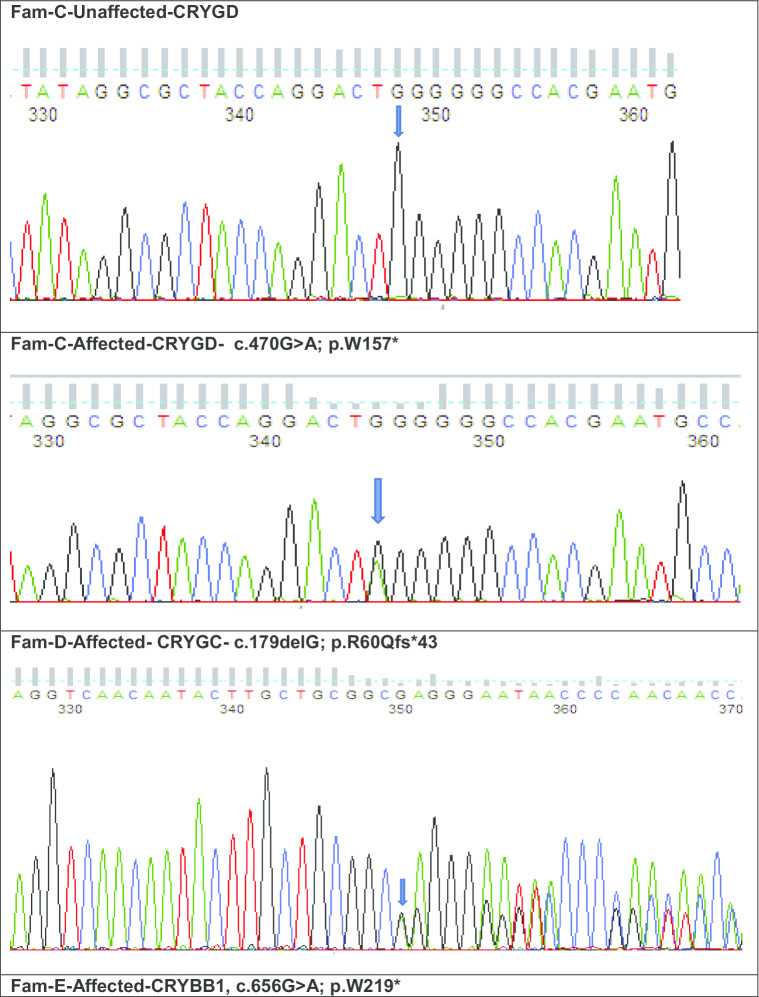

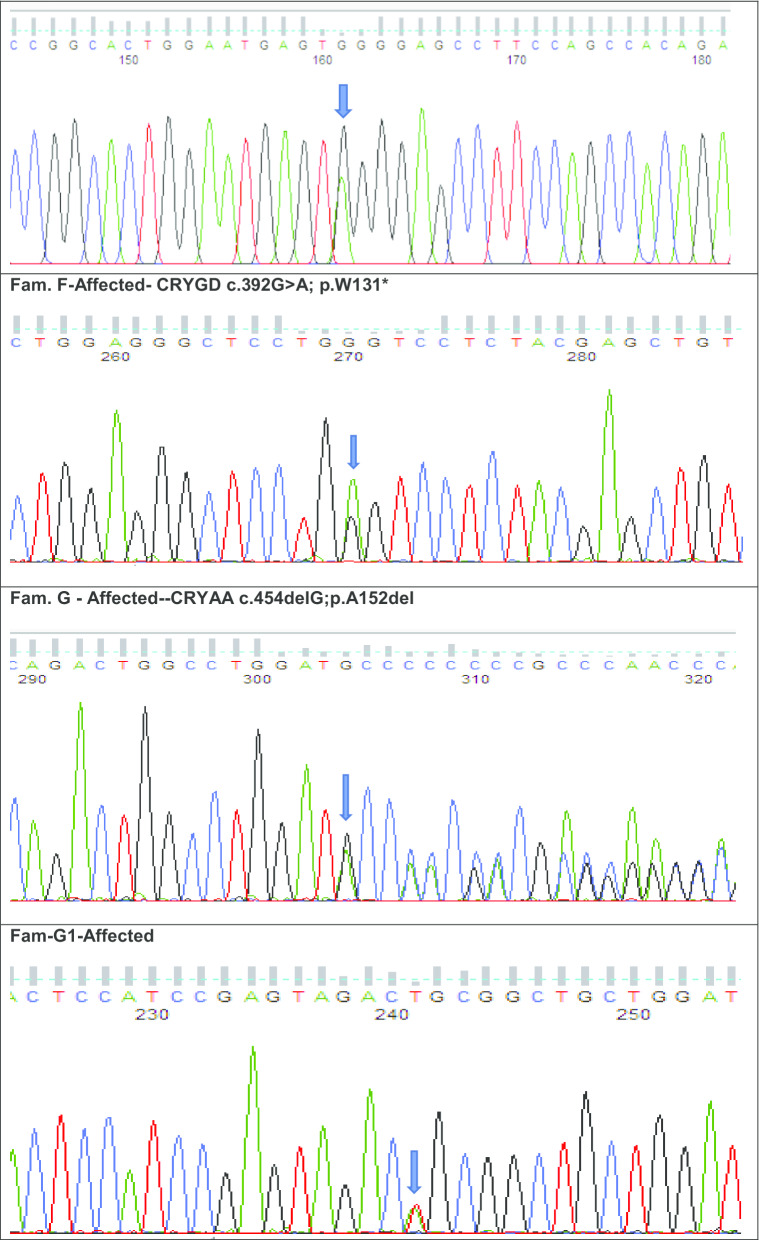

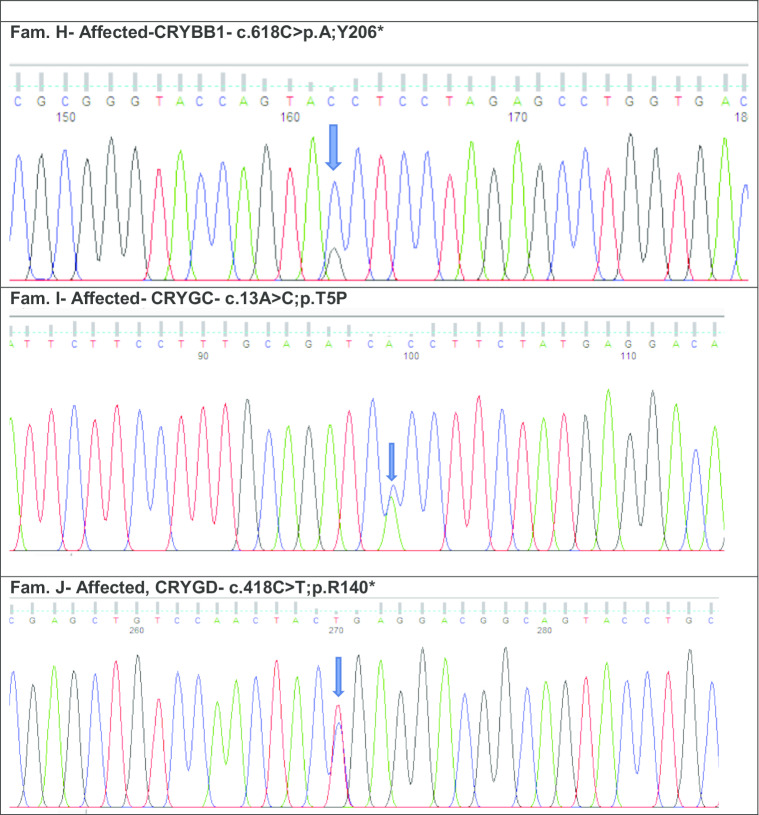
Sequence analysis of Crystallin variants: **a** *CRYAA –*wild type and missense variant c.145C>T in unaffected and affected member of family—A with nuclear cataract; **b** *CRYBA1*—an indel variant at c.272delG in an affected member of family B with nuclear cataract; **c** *CRYGD*—wild type in unaffected and stop codon variant c.470G>A in affected member of family—C with pulverulent cataract; **d** *CRYGC*—a frameshift mutation at c.179delG is shown in the affected member of family-D with nuclear cataract; **e** *CRYBB1*—a stop codon variant c.656G>A in an affected member of family-E with lamellar cataract; **f** *CRYGD*– mutant stop codon amino acid at c.392G>A in an affected female with nuclear cataract; **g** *CRYAA*—a mutant indel variant at c.454delG in affected male with congenital cataract and (G1) *CRYGA*—another missense novel disease-causing variant of uncertain significance at c.118A>T in the same individual G; **h** *CRYBB1*—a stop codon mutation at c.618C>A in affected female with congenital cataract; **i** *CRYGC*—a missense variant at c.13A>C in an affected male with congenital cataract and **j** *CRYGD* a stop codon variant at c.418C>T in an affected female with nuclear cataract

Family A was a five-generation pedigree of 29 individuals, with 14 affected, 7 unaffected, and 8 spouses. This family had nuclear and lamellar opacities with prominent sutures and variable severity. The more severe cataracts were 'needled' in early childhood, and the milder cataracts did not require surgery. The milder ones had prominent sutures and very faint lamellar opacities (Fig. 3a). WES was undertaken in one affected individual (IV-9). After the Phenopolis genetic variant analysis pipeline, variants were filtered by allele frequency and from a total of 119,539 variants, 332 variants remained. The top scoring variant for CADD was a rare heterozygous variant NM_000394.4 c.145C>T; p.R49C in exon 1 of *CRYAA,* with a score of 32. Direct sequencing confirmed the variant (Fig. 4a), which co-segregated in all affected family members.

Family B was a four-generation pedigree, including 9 affected, 10 unaffected, and 5 spouses who were examined, and all affected individuals had nuclear cataract (Fig. 3b). One affected individual (IV-8) was sequenced by WES. Variant annotation and filtering yielded a rare heterozygous variant NM_005208.4 c.272delGAG; p.G91del in exon 4 of *CRYBA1,* with a CADD score of 19.47. Direct sequencing confirmed the variant (Fig. 4b), which co-segregated in the affected family members.

Family C was a four-generation pedigree of 9 members, including 5 affected, 2 unaffected, and 2 spouses. All family members were examined, and an isolated pulverulent cataract was seen in all affected members (Fig. 3c). From this family an affected individual (III-2) was sent for WES. After the Phenopolis genetic variant analysis and filtering, the top scoring variant for CADD (score of 39) was a rare variant NM_006891.4 caused by a point mutation in exon 3 of *CRYGD* at c.470G>A, p.W157*. Direct sequencing validated the variant which co-segregated in affected family members (Fig. 4c).

Family D was a three-generation pedigree of 6 members, including 3 affected, 1 unaffected, and 1 spouse. All the family members were examined, and an isolated nuclear cataract was seen in the affected members (Fig. 3d). Individual II-2 had bilateral cataract surgery in early infancy. WES was undertaken in one affected individual (II-2). Variant annotation and filtering yielded a top scoring (score of 32) rare indel variant, NM_020989.4 c.179delG, p.R60Qfs*43 in exon 2 of *CRYGC.* Direct sequencing confirmed the variant (Fig. 4d), was present in all affected family members.

Family E was a three-generation pedigree of 8 members, with 4 affected, 2 unaffected, and 2 spouses. Six family members were examined, and an isolated lamellar cataract was seen in all the affected members (Fig. 3e). Cataract surgery was performed in young adulthood (late 20’s and 30’s). An affected individual (III-1) was sent for WES. After the Phenopolis genetic variant analysis and filtering, a novel nonsense variant NM_001887.4 c.656G>A, p.W219* in exon 6 of *CRYBB1* was found, with a CADD score of 43*.* The variant was validated by direct sequencing (Fig. 4e), and co-segregated with affected family members.

### Individuals

Individual F with a nuclear cataract underwent WES. Following, variant analysis and filtering the top scoring variant (CADD of 40.00) was a novel variant NM_006891.4 c.392G>A, p. W131* in exon 3 of *CRYGD* (Fig. 4f).

Individual G with unspecified congenital bilateral cataract underwent WES. Following, variant analysis and filtering, 2 variants remained. First, a likely pathogenic novel variant (CADD of 15.18) NM_000394.4, c.454delGCC, p.A152del in exon 3 of *CRYAA*. Interestingly the second variant (CADD of 26.00) was also a novel likely disease-causing variant of uncertain significance, NM_014617.4 c.118A>T, p.S40C in exon 2 of *CRYGA* (Fig. 4g).

Individual H from the ADCC panel underwent WES. Variant analysis and further filtering yielded a novel nonsense pathogenic variant (CADD of 38.00) NM_001887.4, c.618C>A, p. Y206* in exon 6 of *CRYBB1* (Fig. 4h).

Individual I from our ADCC panel underwent WES. After filtering for rare variants with allele frequency < 0.0001 in Gnomad and Kaviar, the top scoring variant (CADD of 24.80) was a mutation and was most likely the previously reported pathogenic variant NM_020989.4 c.13A>C, p.T5P in exon 2 of *CRYGC* (Fig. 4i).

Individual J with a nuclear cataract underwent WES. Following, variant analysis and filtering, the top scoring variant (CADD of 36.00) was also most likely a previously reported nonsense variant NM_006891.4 c.418C>T, p. R140* in exon 3 of *CRYGD* (Fig. 4j).

## Discussion

The crystallins were discovered and named nearly 125 years ago by Morner as the main structural proteins of the ocular lens [21]. The lens is a long lived, ever-growing avascular capsulated organ in the body, composed of lens epithelial cells, which differentiate into lens fibers at the equators of the lens [22, 23]. The lens is mainly composed of crystallins, therefore, to maintain its life-long transparency and optical function [6], crystallin organization in the lens is critically important. The crystallins are expressed from the beginning of its embryological development. Alpha-crystallins (CRYAA, CRYAB) are first to appear in the lens placode and later are very highly expressed in the lens fiber cells [24–26]. CRYAB (αB-crystallin) is expressed throughout the mouse lens from E9.5 (25). The expression of β-crystallins (CRYBA1, CRYBB1) increases after birth and so the highest concentrations of these crystallins are usually found in the lens cortex. However, the expression pattern varies among the individual β-crystallins [27]. Mouse studies has shown that the γ-crystallins (CRYGA-CRYGE) are expressed in the primary lens fiber cells and later in the secondary fiber cells, and seem to be absent from the epithelial cells [28, 29]. The expression of *CRYG* genes reaches at its maximum at birth and then declines during the first weeks after birth [30].

The α-, β- and γ-crystallins constitute a large 13 member family of water soluble, structural proteins. The α-crystallins comprise two subunits—αA and αB and both proteins are considered to be molecular chaperones capable of suppressing protein aggregation [31]. They are members of the small heat-shock protein family and are key components of the cellular chaperone machinery [32, 33]. Whlist *CRYAA* is expressed mainly in the lens and sequence variants are linked with recessive and dominant cataracts, CRYAB is stress-inducible and widely expressed in many tissues and therefore its sequence variants are not only associated with congenital cataract, but also withneurological, cardiac and muscular disorders. The βγ-crystallins are characterized by four Greek key motifs arranged in two domains that are crucial for its folding [34]. Mutations that prematurely truncate the Greek key motifs in domain 2 induce the mutant γ-crystallin to form amyloid fibres that form aggregates in the lens fibre cell nuclei, disrupting nuclear function and causing cataract [35]. Sequence variants in βγ-crystallins are usually linked to autosomal dominant cataract [35].

**Fig. 5:**
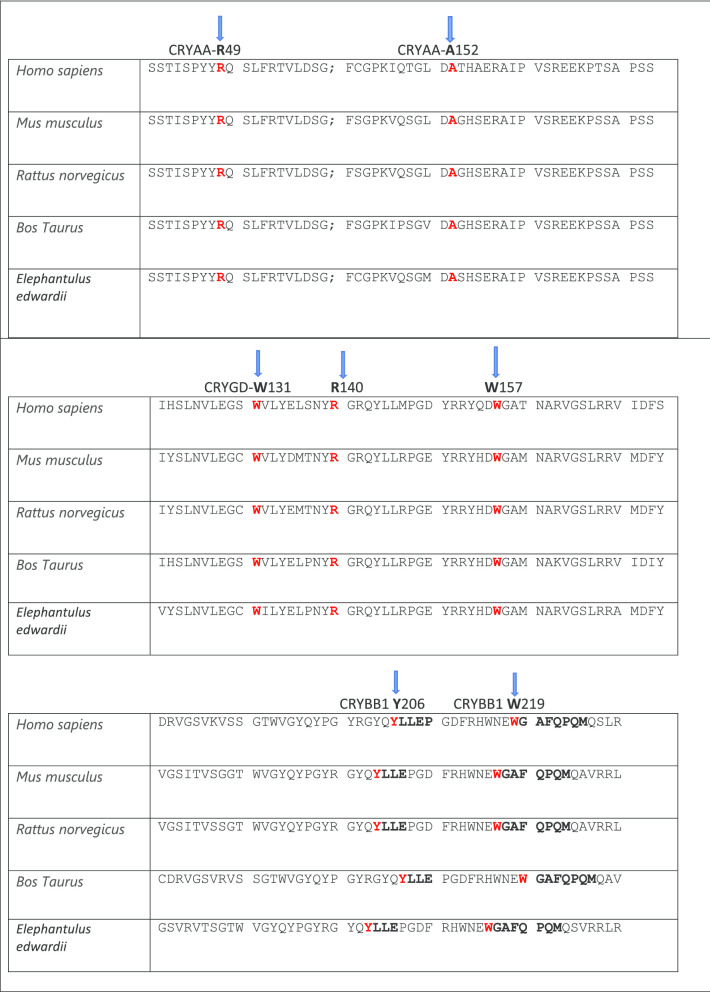

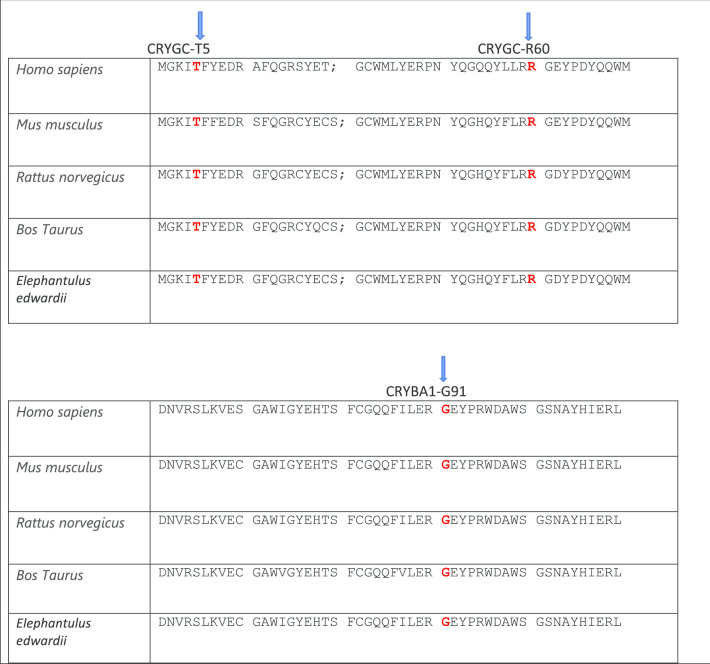
**a** The multiple-sequence alignments from different vertebrate species. Arrows show conserved arginine at p.R49 and alanine at p.A152 in CRYAA protein (https://www.ncbi.nlm.nih.gov/nuccore/?term); **b** The multiple-sequence alignments from different vertebrate species. Arrows show conserved tryptophan at p.W131, p.W157 and arginine at p.R140. in CRYGD protein (https://www.ncbi.nlm.nih.gov/nuccore/?term=Homo+sapiens+CRYGD); **c** The multiple-sequence alignments from different vertebrate species. Arrows show conserved tyrosine at p,Y206 and tryptophan at p.W219 in CRYBB1 protein (https://www.ncbi.nlm.nih.gov/nuccore/?term=human+CRYBB1); **d** The multiple-sequence alignments from different vertebrate species. Arrows show conserved threonine at p,T5 and arginine at p.R60 in CRYGC protein (https://www.ncbi.nlm.nih.gov/nuccore/?term=human+CRYGC); **e** The multiple-sequence alignments from different vertebrate species. Arrows show conserved glycine at p. in CRYBA1 protein (https://www.ncbi.nlm.nih.gov/nuccore/?term=human+CRYBA1)

Here we report 10 heterozygous disease-causing variants in five European families (A-E) and in five isolated individual cases (F–J) of British origin with isolated autosomal dominant congenital cataract (ADCC). All the pathogenic variants found in our families are phylogenetically conserved in the CRYAA, CRYGD, CRYBB1, CRYGC and CRYBA1 proteins (Fig. 5).

### CRYAA

In Family A we have found a recurrent heterozygous variant c.145C>T, which results in an arginine (positively charged) to cysteine (uncharged) substitution at position 49 (R49C), in the first exon of *CRYAA* responsible for an AD congenital nuclear cataract. This variant was first reported by [36]. It resulted in the abnormal localization of the mutant protein to the nucleus and also failed to protect from staurosporine-induced apoptotic cell death [36]. Interestingly, more than 75% of disease-causing variants are located mostly at the N-terminal domain (amino-acid residues 1–63), one in the α-crystallin domain (aa. 64–105) and four in the C-terminal (aa.106–175) of αA-crystallin, comprising R12C, R21W, R21L, R21Q, R49C, R54C, R65Q, R116C, R116H, R117H and R119H; spanning multiple different ethnic groups around the globe causing congenital cataract (CC). This suggests that arginine is likely to be functionally important and therefore mutations in this residue will introduce structural constraints that affect protein function [37]. We have found a second novel heterozygous variant 454delGCC; p.A152del in exon 3, in the C-terminal end of *CRYAA* in Individual G.

### CRYBA1

A rare indel variant, p.G91del, was found in family B, in exon 4 of *CRYBA1* causing an AD congenital nuclear cataract. Previously, Reddy et al., found the p.G91del variant in a large British family with lamellar cataract and demonstrated defective folding and reduced solubility of the mutant protein [38]. Since then, the p.G91del variant has been reported in 14 families of various ethnicity, mostly causing autosomal dominant congenital nuclear or lamellar cataract, except one with esotropia and nystagmus along with congenital cataract [39].

### CRYGD

Family C and individuals F and J harboured three different nonsense variants in *CRYGD*. In family C, a rare heterozygous variant at c.470G>A responsible for an AD congenital pulverulent cataract, resulted in a premature translation stop codon at position W157, located in the cytoplasmic carboxy-terminal region of the CRYGD protein. Previously, the same variant was reported to cause central nuclear cataract in a family of Indian origin [40] and in a Chinese family with isolated congenital cataract [41]. We have reported this variant for the first time in a European population with a different phenotype. A novel heterozygous variant in individual F, at c.392G>A in exon 3 caused a nuclear cataract, due to a truncated protein at p.131aa located in the C-terminal region of the CRYGD protein. Another heterozygous variant in individual J, at c.418C>T in exon 3, also resulted in a premature stop codon in a highly conserved arginine at position p.R140X of CRYGD, causing congenital nuclear cataract. Interestingly, this p.R140X variant has been seen in one family of Chinese origin with nuclear and posterior cataract phenotype [42], and one sporadic Chinese isolated case with nystagmus and total cataract [43]. The same sequence variant has also been seen in a Jewish family and in a family from Indian origin, both exhibiting nuclear cataract [44, 45].

Nearly one-third of pathogenic variants have been found in exon 3, resulting in frameshifts (seven) and stop codon (fourteen), mostly responsible for nuclear or total cataract. The predicted consequence of both the frameshift and premature stop codons will be to truncate the third and also remove the 4th Greek key motif. This will completely change the folding of domain 2 in these γ-crystallins leading to amyloid fibre formation and aggregates in the nuclei of lens fibre cells [35]. Another effect of this class of mutation is to dramatically alter the distribution of the cytoskeletal protein, BFSP2 from the cytoplasm to the nucleus and preventing the transcription factor, Prox1 from accumulating in the same nuclei as described by [35]. These mutations will also abrogate the oxidoreductase activity recently discovered to be associated with CRGYD [46, 47]. Therefore, these variants altered multiple facets of fibre cell differentiation, leading to nuclear cataract in the mouse models where these were first described [48]. One-third of disease-causing variants been found at c.70C>A; p. Pro24Thr, mainly displaying coralliform phenotype in families of different ethnicity and from different continents.

### CRYGC

In family D and in individual I, we identified two different heterozygous variants in *CRYGC*. The novel p.Arg60Glnfs*43 frameshift variant identified in family D resulted from a guanine deletion that introduced a premature translation stop codon located in the N-terminal region of CRYGC protein, and associated with nuclear cataract. It is conceivable that this mutation will affect CRYGC, in a similar fashion to the impact of CRYGD mutations described above, given that this would form a truncated domain 1, with only the first Greek key being complete. It is highly likely that any protein product will be unstable because of the missing second Greek key which is needed to stabilise the other Greek key in domain 1. This domain is anyway more unstable than domain 2 and both have amyloid forming potential [49–51]. It is therefore likely that the mutant protein will also form amyloid fibres. Individual I had a known likely pathogenic variant p.T5P, causing bilateral congenital cataract. This variant was previously reported by Heon et al. 1999 in a British family with central zonular pulverulent cataract [52]. Sequence variants in *CRYGC* have been associated with nuclear and lamellar cataract [53], along with additional eye anomalies such as glaucoma, microcornea [54, 55], microphthalmia [56] and optic disc coloboma [57]. These phenotypic variations could be due to as yet unidentified modifier genes.

### CRYBB1

Family E and individual H harboured two heterozygous nonsense variants in exon 6 of *CRYBB1*. In family E, a novel heterozygous variant at c.656G>A responsible for an AD congenital lamellar cataract, resulted in a premature translation stop codon at p.W219, located in the cytoplasmic carboxy-terminal region of CRYBB1. This mutation will also severely disrupt the fourth and final Greek key motif, which is needed to stabilise the whole domain [58]. It remains to be proven whether the β-crystallins have amyloid forming potential similar to the γ-crystallins [35, 49–51]. CRYBB1 is also expressed in tissues other than the eye lens and its altered expression has been reported to be potentially associated with schizophrenia [59].This has led to the fascinating proposal that neurological disorders and eye disease, such as cataract, may have a common cause [60]; with the link to protein amyloid formation certainly adding weight to this proposal. Interestingly, individual H with an isolated bilateral CC also had a novel pathogenic stop codon variant at c.618C>A; p.Y206*, close to p.W219*. It is of note that the majority of disease-causing variants located at the N-terminal end of the protein display recessive inheritance, while the variants in the C-terminal region generally exhibit dominant inheritance. This perhaps reflects a role for nonsense-mediated mRNA decay in the inheritance pattern.

## Conclusions

We report 5 novel and 5 recurrent disease-causing variants in the Crystallins causing inherited congenital cataract, and comprehensively review the genetic landscape in the Crystallin genes. Our study further extends the mutation spectrum associated with the Crystallin genes and further facilitates clinical diagnosis. We highlight the amyloid-forming potential of the mutated βγ-crystallins as a common mechanistic basis to the novel mutations reported here and the potential multimorbidity of crystallin mutations with neurological disorders.

Many of the identified variants causing isolated cataract in this study provide further evidence of phenotypic heterogeneity and further showcase the significance of merging clinical observation with NGS, in order to understand the biological basis for phenotypic variation associated with familial cataract as a valuable paradigm to understand the genetic basis of human disease.

The clinical and genetic heterogeneity now reported in congenital cataract has begun to rival the vast variability documented in inherited retinal disease; making ophthalmic genetics the most heterogeneous in Medicine.

## Data Availability

The datasets used and/or analysed during the current study are available from the corresponding author on reasonable request.
